# Supplementary material for: Structural serology of polyclonal antibody responses to mRNA-1273 and NVX-CoV2373 COVID-19 vaccines
Source: bioRxiv. 2024 Dec 12:2024.12.11.628030. Preprint. [Version 1] doi: 10.1101/2024.12.11.628030 (PMC11661243; doi:10.1101/2024.12.11.628030)
Supplement: Supplement 1 [file media-1.pdf]

Supplementary Materials

Structural serology of polyclonal antibody responses to mRNA-1273 and NVX-CoV2373 COVID-19 vaccines

Sandhya Bangaru[1, #], Abigail M. Jackson[1, #], Jeffrey Copps[1], Monica L. Fernández-Quintero[1], Jonathan L. Torres[1], Sara T. Richey[1], Bartek Nogal[1], Leigh M. Sewall[1], Alba Torrents de la Peña[1], Asma Rehman[2], Mimi Guebre-Xabier[2], Bethany Girard[3], Rituparna Das[3], Kizzmekia S. Corbett-Helaire[4,5,6], Robert A. Seder[4], Barney S. Graham[4,7], Darin K. Edwards[3], Nita Patel[2], Gale Smith[2], Andrew B. Ward[1, *]

[1]Dept. of Integrative Structural and Computational Biology, The Scripps Research Institute; La Jolla, CA, 92037, USA
[2]Novavax, Inc; 21 Firstfield Road, Gaithersburg, MD, 20878, USA
[3]Moderna, Inc; Cambridge, MA, 02142, USA
[4]Vaccine Research Center; National Institutes of Allergy and Infectious Diseases; National Institutes of Health; Bethesda, Maryland, 20892, USA
[5]Current affiliation: Department of Immunology and Infectious Diseases; Harvard T.H. Chan School of Public Health; Boston, Massachusetts, 02115, USA
[6]Current affiliation: Howard Hughes Medical Institute; Chevy Chase, Maryland, 20815, USA
[7]Current affiliation: Department of Microbiology, Biochemistry & Immunology; Morehouse School of Medicine; Atlanta, Georgia, 30310, USA
[#]These authors contributed equally
***Correspondence:**
Andrew B. Ward

Figures

A.

Animal ID	D35 Anti-rS (EC50)	D35 Plaque Reduction Neutralization Titers (PRNT50) USA-WA1/2020
NVXWu-NHP1	62222	1673.29
NVXWu-NHP2	168573	3568.71
NVXWu-NHP3	33412	1074.24
NVXWu-NHP4	327608	4371.4

B.

Animal ID	ID50 Titer (Week6)
Mod-NHP1	3323
Mod-NHP2	4036
Mod-NHP3	7299
Mod-NHP4	8609

C.

Pooled Groups	Animal ID	D35 Anti-rS Wuhan (EC50)	D35 Anti-rS SA (EC50)	D35 SARS-CoV-2 Plaque Reduction Neutralization Titers (PRNT50)			
				USA-WA1/2020	Beta B.1.351	Alpha B.1.1.7	Delta B.1.617.1
Homologous NVX-CoV2373 prime-boost	NVXWu-NHP1	62222	40288	1673.29	650.25	4684.25	4926.17
	NVXWu-NHP2	168573	97100	3568.71	2097.99	8608.69	5201.07
	NVXWu-NHP3	33412	21991	1074.24	324.86	1010.4	1349.95
	NVXWu-NHP4	327608	235987	4371.4	4802.9	11525.65	22316.01
	NVXWu-NHP5	181272	121172	4478.01	3104.66	12750.32	17762.63
Homologous rS-B.1.351 prime-boost	NVXSA-NHP1	24418	51650	433.96	2640.67	2262.67	993.9
	NVXSA-NHP2	176122	309877	504.45	14752.85	1732.13	1168.25
	NVXSA-NHP3	83828	172131	404.07	6187.01	2828.59	346.84
	NVXSA-NHP4	31414	60845	303.1	4167.7	1278.43	614.93
	NVXSA-NHP5	235781	448973	1318.11	22091.49	9236.44	4289.18
Heterologous NVX-CoV2373/rS-B.1.351 prime-boost	NVXWu/SA-NHP1	83327	81397	1156.28	587	2066.27	517.68
	NVXWu/SA-NHP2	123916	129238	1574.24	2642.11	4383.54	411.63
	NVXWu/SA-NHP3	275603	234235	3295.05	4594.49	6069.98	845.41
	NVXWu/SA-NHP4	183030	167035	3736.96	5152.95	7773.29	2720.84

Figure S1. NHP serum antibody responses to NVX-CoV2373, NVX rS-Beta, and mRNA-1273 (A) Day 35 serum binding titers to recombinant Wuhan Spike shown as half-maximal effective concentration (EC_{50}) and WA1/2020 virus inhibition titers indicated as half-maximal plaque reduction neutralization titers ($PRNT_{50}$) from NHPs immunized with NVX-CoV2373 prime and boost. **(B)** Week 6 serum neutralizing titers shown as 50% inhibitory dose (ID_{50}) from NHPs immunized with mRNA-1273 prime-boost regimen. **(C)** Day 35 serum binding titers to recombinant Spikes (rS) from Wuhan and Beta (SA) strains shown as half-maximal effective

concentrations (EC_{50}) and virus neutralization titers against WA1/2020, Beta B.1.351, Alpha B.1.1.7, and Delta B.1.617.1 viruses indicated as half-maximal plaque reduction neutralization titers ($PRNT_{50}$) from NHPs immunized with either homologous NVX-CoV2373 or rS-Beta prime and boost series or heterologous NVX-CoV2373 prime and rS-Beta boost. Samples highlighted in grey are the same samples as in (A). Sample highlighted in yellow was used for cryo-EM studies.

NVX-CoV2373	NVX-Wu NHP1 + Wu Spike Mix	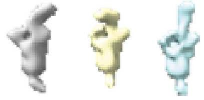
	NVX-Wu NHP2 + Wu Spike Mix	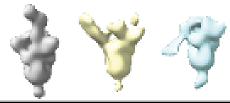
	NVX-Wu NHP3 + Wu Spike Mix	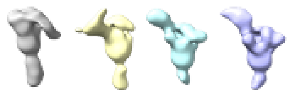
	NVX-Wu NHP4 + Wu Spike Mix	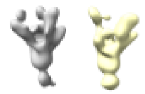
mRNA-1273	Mod NHP1 + Wu Spike Mix	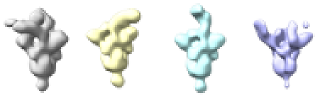
	Mod NHP2 + Wu Spike Mix	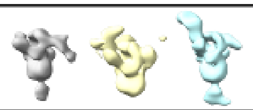
	Mod NHP3 + Wu Spike Mix	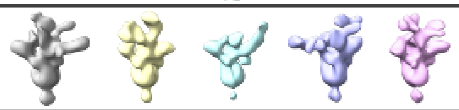
	Mod NHP4 + Wu Spike Mix	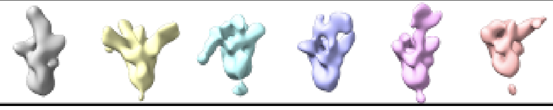
NVX-CoV2373	NVX-Donor2 (Day 21) + Wu Spike Mix	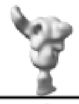
	NVX-Donor3 (Day 21) + Wu Spike Mix	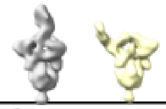
	NVX-Donor4 (Day 21) + Wu Spike Mix	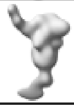
	NVX-Donor1 (Day 49) + Wu Spike Mix	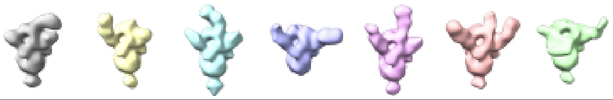
	NVX-Donor2 (Day 49) + Wu Spike Mix	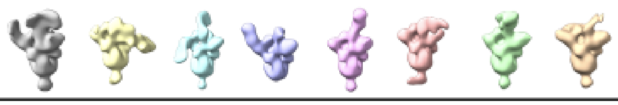
	NVX-Donor3 (Day 49) + Wu Spike Mix	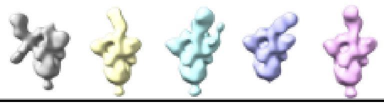
	NVX-Donor4 (Day 49) + Wu Spike Mix	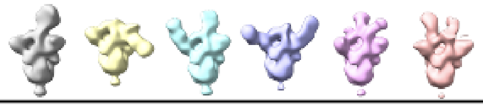
	NVX-Donor5 (Day 49) + Wu Spike Mix	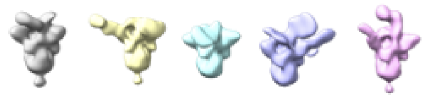

NVX-CoV2373	NVX-Donor1 (Day 105) + Wu Spike Mix	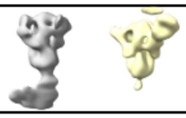
	NVX-Donor2 (Day 105) + Wu Spike Mix	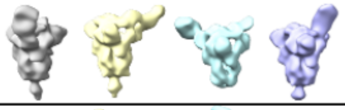
	NVX-Donor3 (Day 105) + Wu Spike Mix	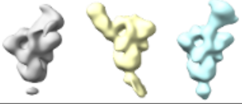
	NVX-Donor4 (Day 105) + Wu Spike Mix	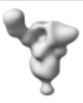
mRNA-1273	Mod-Donor1 (Day 29) + Wu Spike Mix	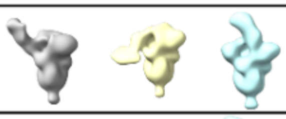
	Mod-Donor2 (Day 29) + Wu Spike Mix	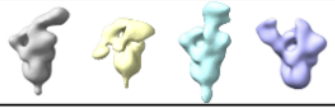
	Mod-Donor3 (Day 29) + Wu Spike Mix	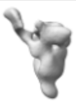
	Mod-Donor4 (Day 29) + Wu Spike Mix	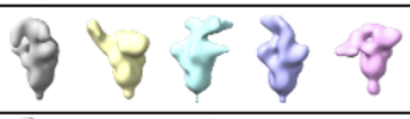
	Mod-Donor1 (Day 43) + Wu Spike Mix	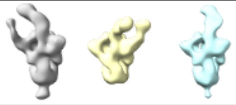
	Mod-Donor2 (Day 43) + Wu Spike Mix	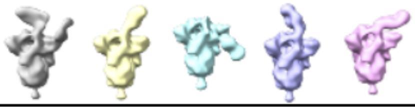
	Mod-Donor3 (Day 43) + Wu Spike Mix	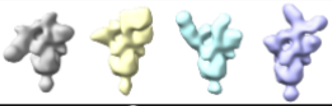
	Mod-Donor4 (Day 43) + Wu Spike Mix	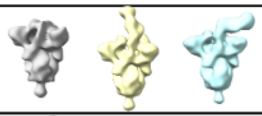
NVX-CoV2373	NVX-Wu NHP1 + Alpha Variant Spike	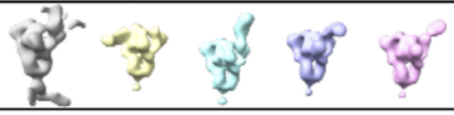
	NVX-Wu NHP1 + Beta Variant Spike	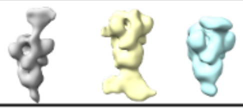

NVX-CoV2373	NVX-Wu NHP2 + Alpha Variant Spike	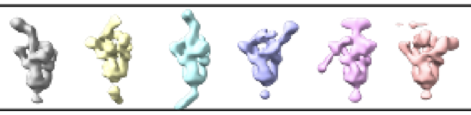
	NVX-Wu NHP2 + Beta Variant Spike	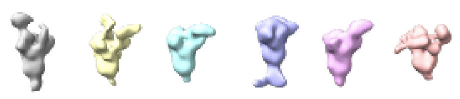
	NVX-Wu NHP2 + Delta Variant Spike	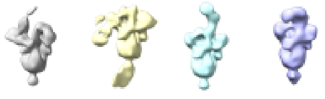
	NVX-Wu NHP2 + Omicron Variant Spike	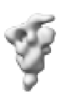
mRNA-1273	Mod NHP4 + Alpha Variant Spike	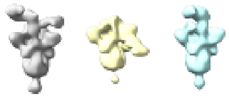
	Mod NHP4 + Beta Variant Spike	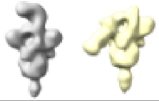
	Mod NHP4 + Delta Variant Spike	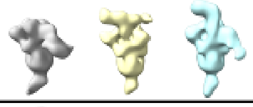
	Mod NHP4 + Omicron Variant Spike	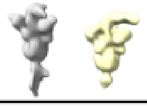
	Mod NHP1 + Alpha Variant Spike	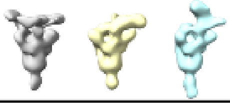
	Mod NHP1 + Beta Variant Spike	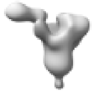
	Mod NHP1 + Delta Variant Spike	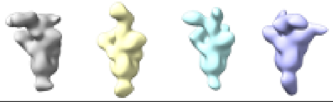
	Mod NHP1 + Omicron Variant Spike	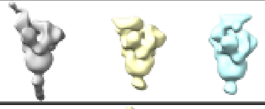
NVX-CoV2373	NVX- D49 Donor Pool + Ancestral Spike	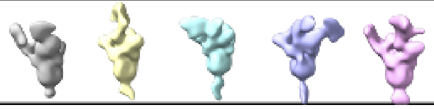
	NVX- D49 Donor Pool + Beta Variant Spike	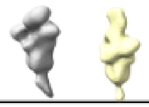
	NVX- D49 Donor Pool + Delta Variant Spike	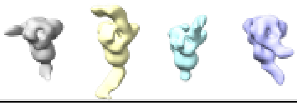
	NVX- D49 Donor Pool + Omicron Variant Spike	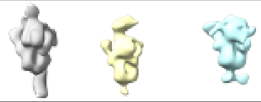

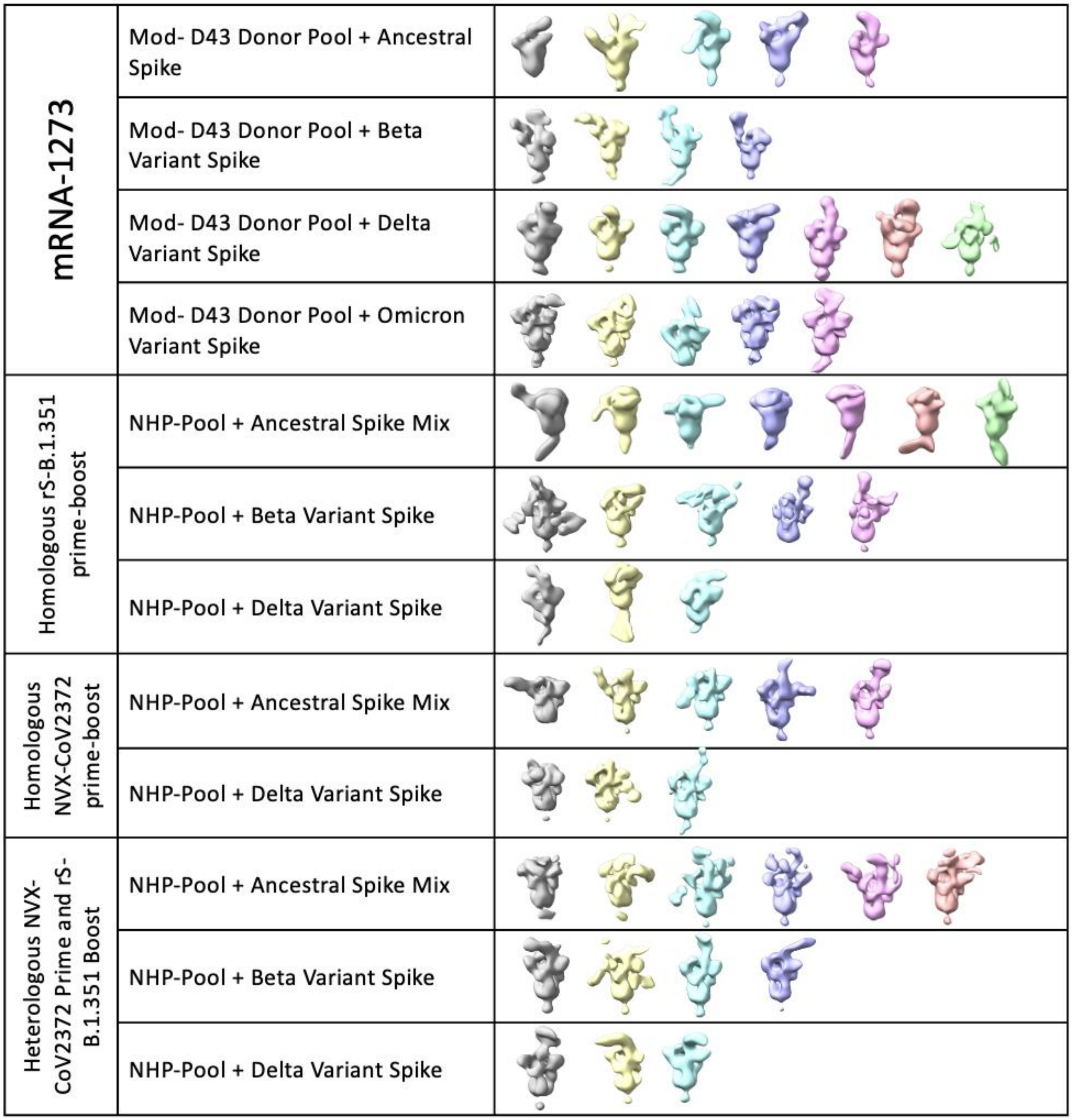

Figure S2. Negative stain-EM 3D classes from each dataset used for generation of composite maps displaying all the specificities.

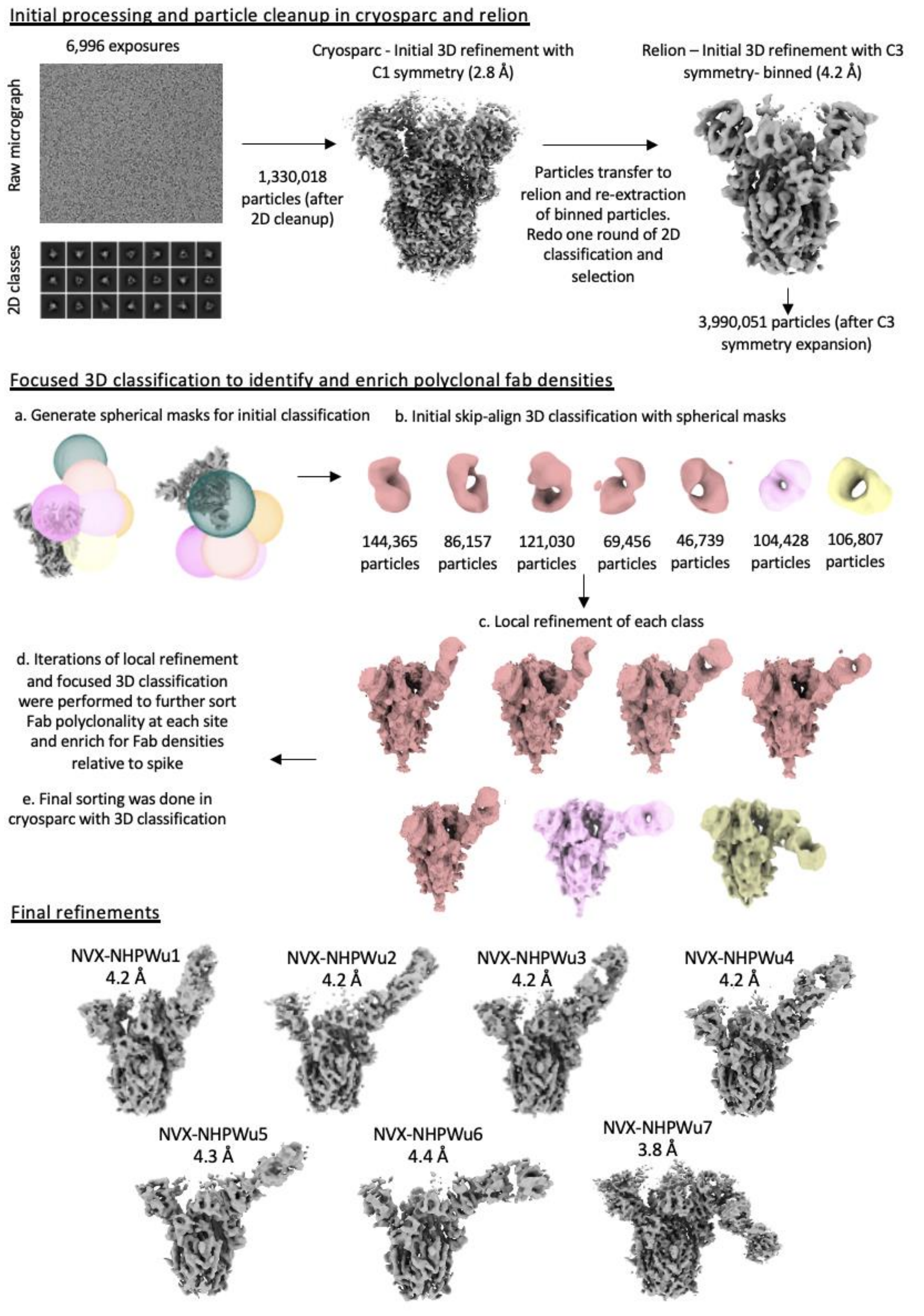

Figure S3. Schematic representation of the cryo-EMPEM processing workflow for pooled NVX-CoV2373 prime-boosted NHP Fabs complexed with SARS-CoV-2-HP-GSAS-Mut7-

D614G Spike. The focused classification approach used for generating Fab-Spike reconstructions is shown in steps.

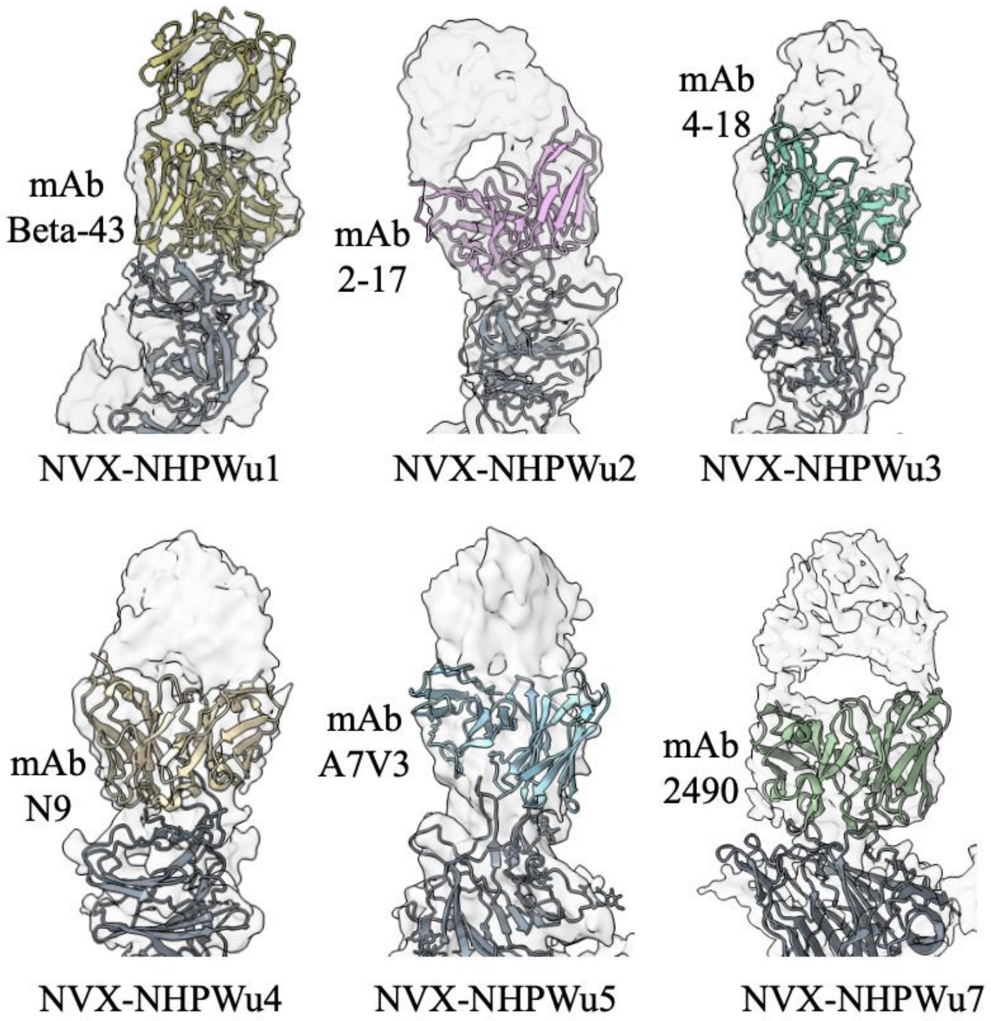

Figure S4. Published monoclonal Spike-NTD antibodies resembling NVX-NHPWu pAbs. Atomic models of NTD in complex with mAbs Beta-43 (PDB# 7Q91), 2-17 (PDB# 7LQW), 4-18 (PDB# 7L2E), N9 (PDB# 7E8F), A7V3 (PDB# 7SJ0), and 2490 (PDB# 7DZY) docked into cryo-EMPEM map densities (transparent) of pAbs NVX-NHPWu1, NVX-NHPWu2, NVX-NHPWu3, NVX-NHPWu4, NVX-NHPWu5, and NVX-NHPWu7, respectively.

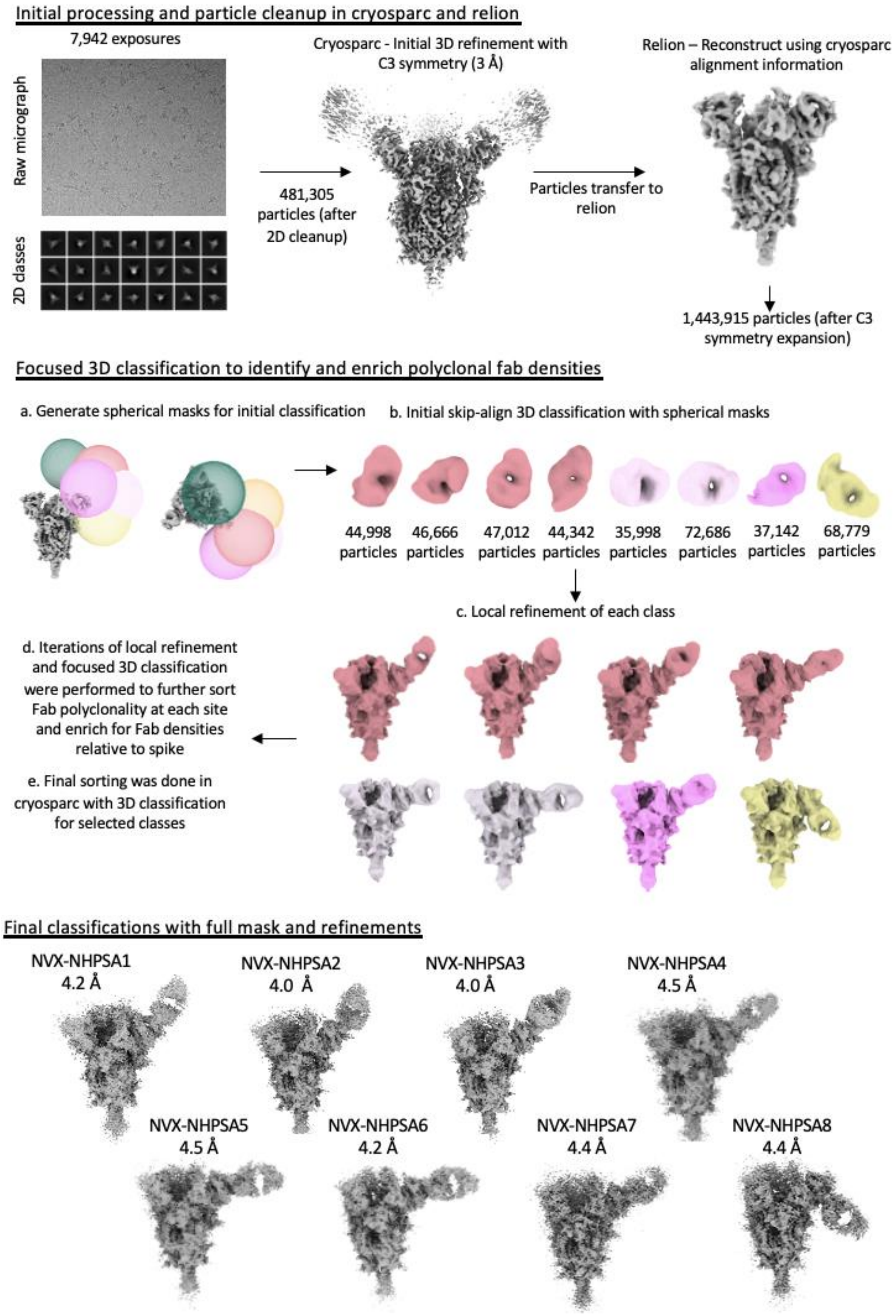

Initial processing and particle cleanup in cryosparc and relion
7,942 exposures
Raw micrograph
2D classes
481,305 particles (after 2D cleanup)
Cryosparc - Initial 3D refinement with C3 symmetry (3 Å)
Particles transfer to relion
Relion – Reconstruct using cryosparc alignment information
1,443,915 particles (after C3 symmetry expansion)
Focused 3D classification to identify and enrich polyclonal fab densities
a. Generate spherical masks for initial classification
b. Initial skip-align 3D classification with spherical masks
44,998 particles
46,666 particles
47,012 particles
44,342 particles
35,998 particles
72,686 particles
37,142 particles
68,779 particles
c. Local refinement of each class
d. Iterations of local refinement and focused 3D classification were performed to further sort Fab polyclonality at each site and enrich for Fab densities relative to spike
e. Final sorting was done in cryosparc with 3D classification for selected classes
Final classifications with full mask and refinements
NVX-NHPSA1 4.2 Å
NVX-NHPSA2 4.0 Å
NVX-NHPSA3 4.0 Å
NVX-NHPSA4 4.5 Å
NVX-NHPSA5 4.5 Å
NVX-NHPSA6 4.2 Å
NVX-NHPSA7 4.4 Å
NVX-NHPSA8 4.4 Å

Figure S5. Schematic representation of the cryo-EMPEM processing workflow for SARS-CoV-2-HP-GSAS-Mut7-D614G Spike complexed with pAbs isolated from a single NHP immunized with NVX-CoV2373/rS-Beta prime-boost.

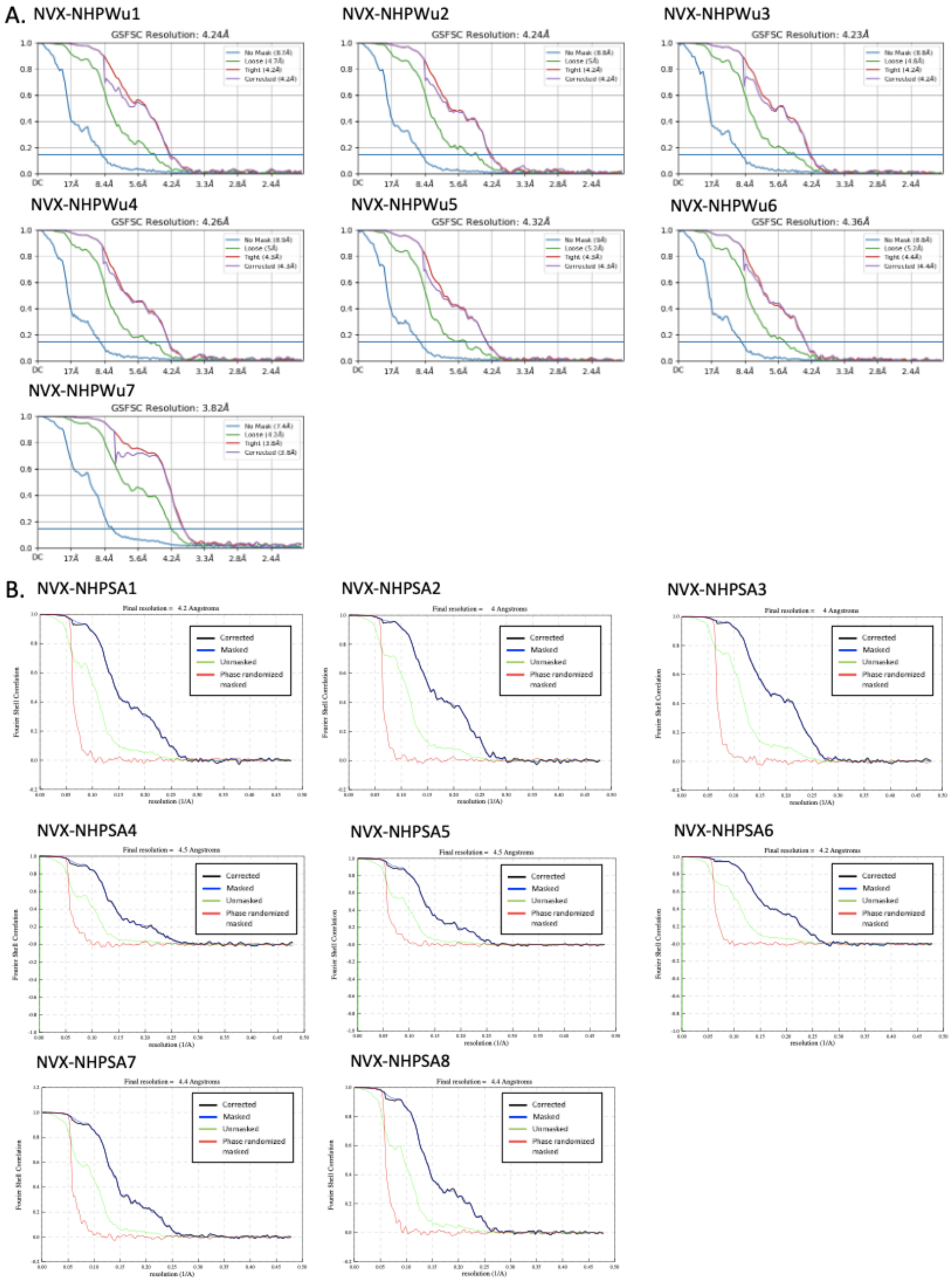

Figure S6. FSC curves for SARS-CoV-2-HP-GSAS-Mut7-D614G Spike-Fab cryo-EMPEM reconstructions from NHPs that received (A) homologous NVX-CoV2373 or (B) heterologous NVX-CoV2373/rS-Beta prime-boost immunizations.

A.

Subject ID	Day	ELISA (IgG Titer)	ACE-2 Inhibition (Ab titer)	Micro neutralization (1/Dilution)
NVX-Donor1	21	3.62E+03	13.5	160
NVX-Donor2	21	7.05E+03	5	80
NVX-Donor3	21	2.22E+04	5	480
NVX-Donor4	21	3.43E+03	12.9	240
NVX-Donor5	21	4.06E+03	5	80
NVX-Donor1	49	8.75E+04	173.1	7680
NVX-Donor2	49	1.00E+05	288.2	10240
NVX-Donor3	49	1.08E+05	184.6	5120
NVX-Donor4	49	1.44E+05	435.3	15360
NVX-Donor5	49	9.66E+04	344.9	5120
NVX-Donor1	105	1.64E+04	33.8	NA
NVX-Donor2	105	5.18E+04	64.4	NA
NVX-Donor3	105	2.28E+04	14.8	NA
NVX-Donor4	105	4.58E+04	155.2	NA
NVX-Donor5	105	2.97E+04	77.5	NA

B.

Subject ID	Day	Endpoint spike titer	Endpoint RBD titer	Pseudoneut titers
Mod-Donor1	29	1.06E+05	8.71E+04	21
Mod-Donor2	29	9.54E+04	9.21E+04	14
Mod-Donor3	29	9.23E+04	1.45E+05	22
Mod-Donor4	29	1.69E+05	1.06E+05	69
Mod-Donor1	43	5.57E+05	3.37E+05	271
Mod-Donor2	43	7.08E+05	5.42E+05	361
Mod-Donor3	43	7.11E+05	3.97E+05	320
Mod-Donor4	43	8.28E+05	7.17E+05	697

Figure S7. Clinical trial donor serum antibody responses to NVX-CoV2373 and mRNA-1273 at pre-boost and post-boost time points (A) Day 21, day 49 and day 105 serum binding titers to recombinant Wuhan Spike shown as half-maximal effective concentration (EC_{50}), ACE2

inhibition titers and wild-type WA1/2020 microneutralization titers indicated as inhibitory concentration of >99% (MN $IC_{>99\%}$) from donors who received NVX-CoV2373 prime and boost. **(B)** Day 29 and day 43 serum binding titers to recombinant Wuhan Spike and recombinant RBD shown as half-maximal effective concentration (EC_{50}), and Wuhan pseudovirus neutralization titers from donors who received mRNA-1273 prime and boost.

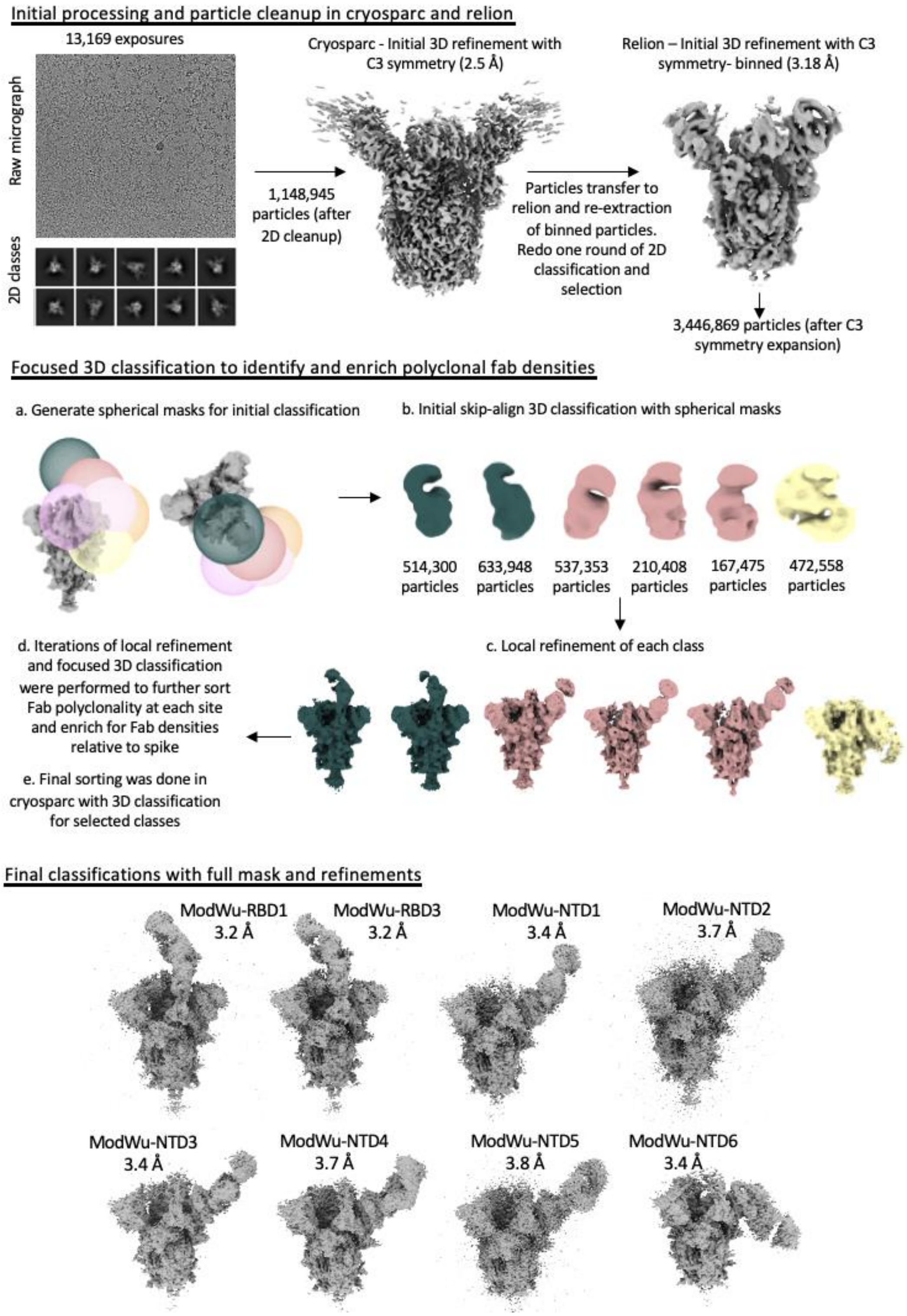

Initial processing and particle cleanup in cryosparc and relion
13,169 exposures
Raw micrograph
2D classes
Cryosparc - Initial 3D refinement with C3 symmetry (2.5 Å)
1,148,945 particles (after 2D cleanup)
Relion – Initial 3D refinement with C3 symmetry- binned (3.18 Å)
Particles transfer to relion and re-extraction of binned particles. Redo one round of 2D classification and selection
3,446,869 particles (after C3 symmetry expansion)
Focused 3D classification to identify and enrich polyclonal fab densities
a. Generate spherical masks for initial classification
b. Initial skip-align 3D classification with spherical masks
514,300 particles
633,948 particles
537,353 particles
210,408 particles
167,475 particles
472,558 particles
c. Local refinement of each class
d. Iterations of local refinement and focused 3D classification were performed to further sort Fab polyclonality at each site and enrich for Fab densities relative to spike
e. Final sorting was done in cryosparc with 3D classification for selected classes
Final classifications with full mask and refinements
ModWu-RBD1 3.2 Å
ModWu-RBD3 3.2 Å
ModWu-NTD1 3.4 Å
ModWu-NTD2 3.7 Å
ModWu-NTD3 3.4 Å
ModWu-NTD4 3.7 Å
ModWu-NTD5 3.8 Å
ModWu-NTD6 3.4 Å

Figure S8. Schematic representation of the cryo-EMPEM processing workflow for pooled mRNA-1273 vaccinated donor serum pAbs (day 43) complexed with SARS-CoV-2-HP-GSAS-Mut7-D614G Spike.

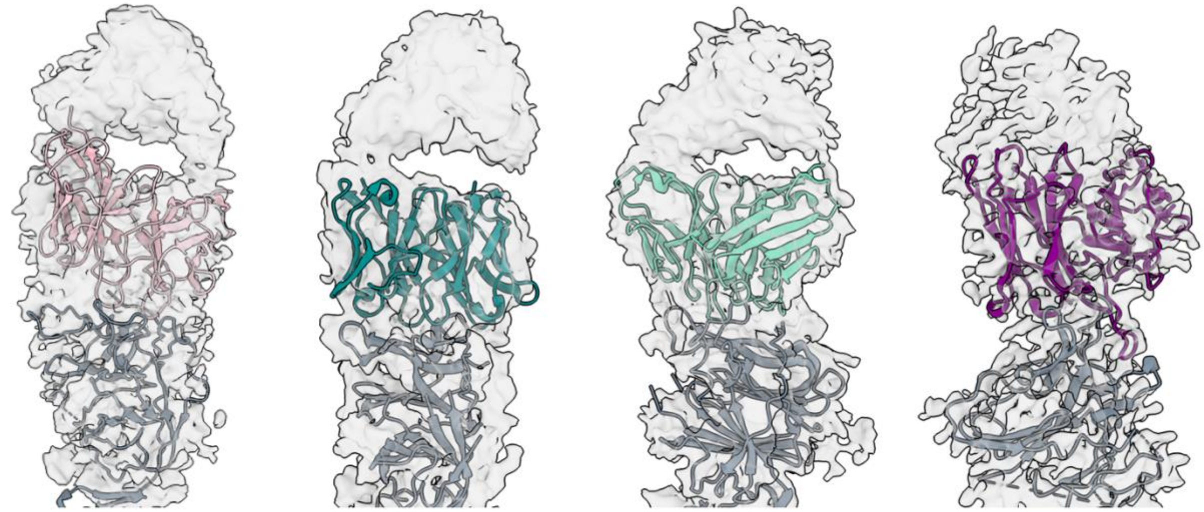

Figure S9. Published monoclonal Spike-NTD antibodies resembling ModWu-NTD pAbs. Atomic models of NTD in complex with mAbs 2-17 (PDB# 7LQW), mAb S2M28 (PDB# 7LY3), 4-18 (PDB# 7L2E), and TXG-0078 (PDB# 8SWH) docked into cryo-EMPEM map densities (transparent) of pAbs ModWu-NTD2, ModWu-NTD3, ModWu-NTD4, and ModWu-NTD5, respectively.

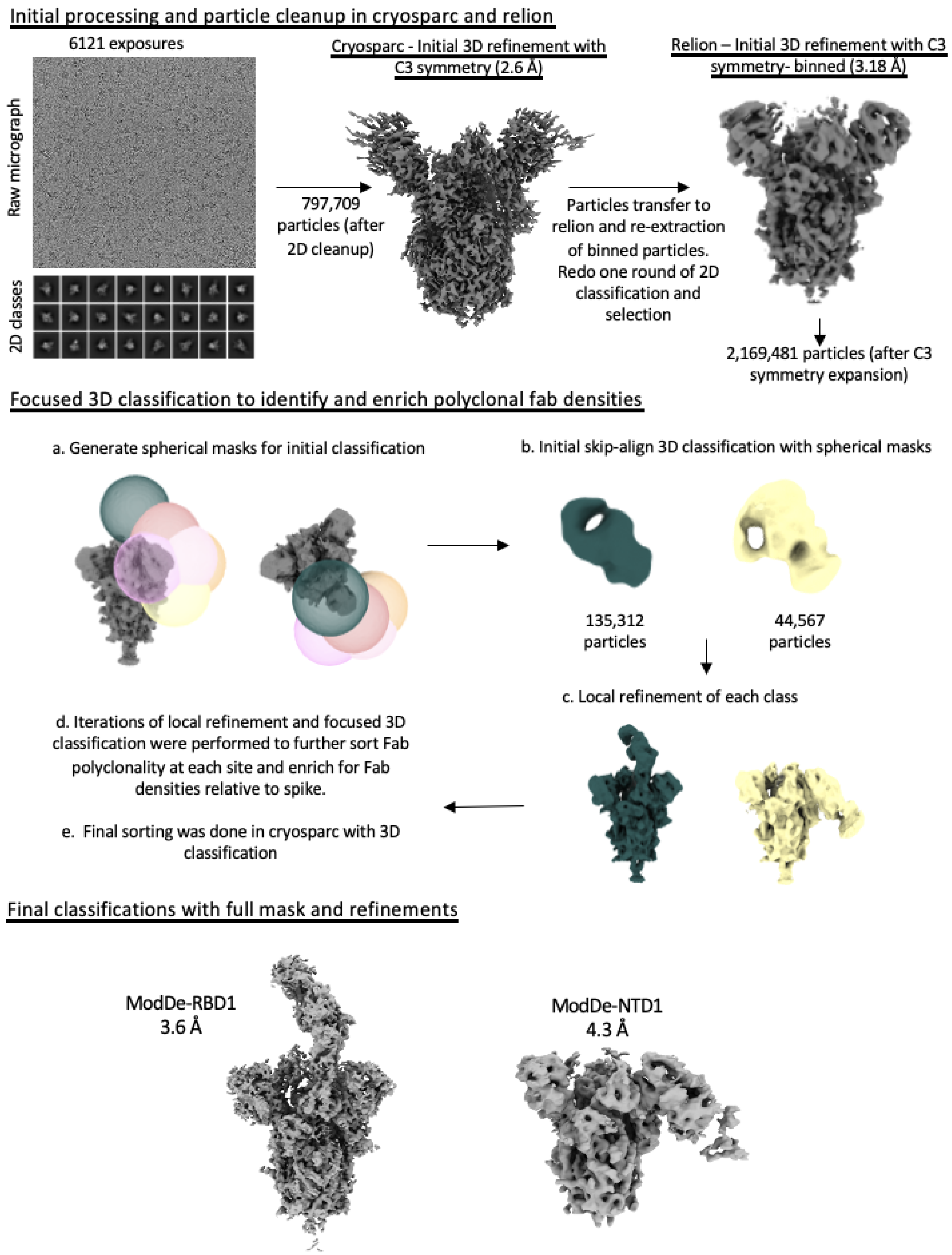

Initial processing and particle cleanup in cryosparc and relion
6121 exposures
Raw micrograph
2D classes
797,709 particles (after 2D cleanup)
Cryosparc - Initial 3D refinement with C3 symmetry (2.6 Å)
Particles transfer to relion and re-extraction of binned particles. Redo one round of 2D classification and selection
Relion – Initial 3D refinement with C3 symmetry- binned (3.18 Å)
2,169,481 particles (after C3 symmetry expansion)
Focused 3D classification to identify and enrich polyclonal fab densities
a. Generate spherical masks for initial classification
b. Initial skip-align 3D classification with spherical masks
135,312 particles
44,567 particles
c. Local refinement of each class
d. Iterations of local refinement and focused 3D classification were performed to further sort Fab polyclonality at each site and enrich for Fab densities relative to spike.
e. Final sorting was done in cryosparc with 3D classification
Final classifications with full mask and refinements
ModDe-RBD1
3.6 Å
ModDe-NTD1
4.3 Å

Figure S10. Schematic representation of the cryo-EMPEM processing workflow for pooled mRNA-1273 vaccinated donor serum pAbs (day 43) complexed with SARS-CoV-2 Delta-HP-Mut7 Spike.

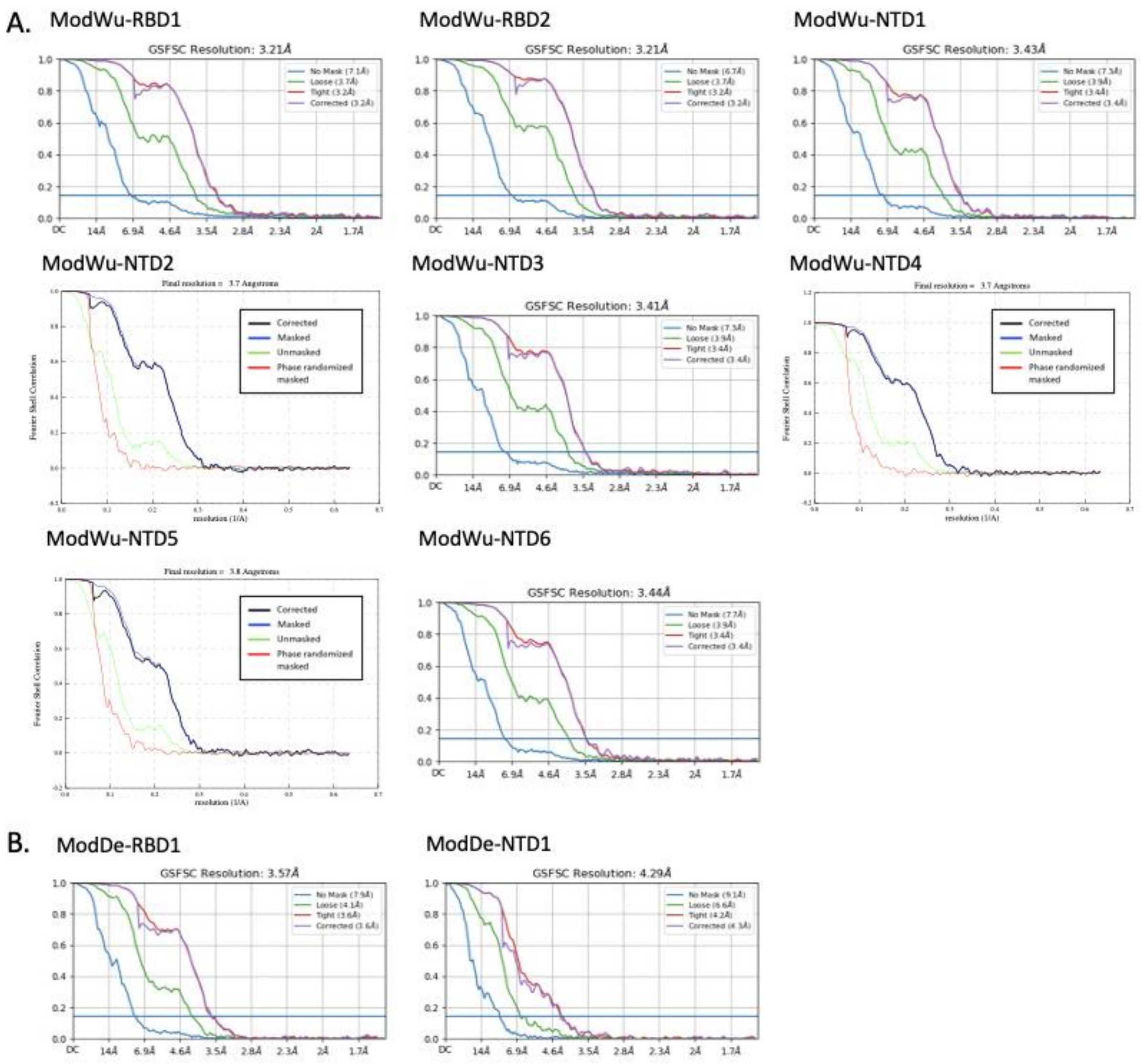

Figure S11. FSC curves for mRNA-1273 vaccinated donor serum pAbs (day 43) complexed with (A) SARS-CoV-2-HP-GSAS-Mut7-D614G or (B) SARS-CoV-2 Delta-HP-Mut7 Spike

Table S1. Cryo-EM data collection

**Data collection**	**NVX-NHPWu pAbs complexed with SARS-CoV-2-HP-GSAS-Mut7-D614G Spike**	**NVX-NHPSA pAbs complexed with SARS-CoV-2-HP-GSAS-Mut7-D614G Spike (2 sessions)**	**Mod-D49 pAbs complexed with SARS-CoV-2-HP-GSAS-Mut7-D614G Spike**	**Mod-D49 pAbs complexed with SARS-CoV-2 Delta-HP-Mut7 Spike**
Microscope	FEI Titan Krios	FEI Titan Krios	FEI Titan Krios	FEI Titan Krios
Voltage (kV)	300	300	300	300
Detector	Gatan K2 Summit	Gatan K2 Summit	Gatan K3	Gatan K3
Recording mode	Counting	Counting	Super-resolution	Super-resolution
Nominal magnification	130,000	130,000	29,000	29,000
Movie micrograph pixel size (Å)	1.045	1.045	0.788	0.788
Number of frames per movie micrograph (Falcon 4 EER fractions)	35	35	67	67
Total dose (e^-/Å^2)	50.6	50.6/50.1	60	60
Number of movie micrographs	6996	7942	14094	7958

Table S2. Cryo-EMPEM refinement parameters for NVX-NHPWu pAbs

**Map**	**NVX-NHPWu1**	**NVX-NHPWu2**	**NVX-NHPWu3**	**NVX-NHPWu4**	**NVX-NHPWu5**	**NVX-NHPWu6**	**NVX-NHPWu7**
EMDB	EMD-48199	EMD-48200	EMD-48201	EMD-48202	EMD-48203	EMD-48204	EMD-48205
Number of molecular projection images in map	13,248	13,989	12,190	12,140	11,441	13,125	37,096
Symmetry	C1	C1	C1	C1	C1	C1	C1
Map resolution (FSC 0.143; Å)	4.2	4.2	4.2	4.3	4.3	4.4	3.8
Map sharpening B-factor (Å^2)	-55.9	-55.9	-52.4	-52	-46.1	-57.4	-75.8

Table S3. Cryo-EMPEM refinement parameters for NVX-NHPSA pAbs

**Map**	**NVX-NHPSA1**	**NVX-NHPSA2**	**NVX-NHPSA3**	**NVX-NHPSA4**	**NVX-NHPSA5**	**NVX-NHPSA6**	**NVX-NHPSA7**	**NVX-NHPSA8**
EMDB	EMD-48206	EMD-48207	EMD-48208	EMD-48209	EMD-48210	EMD-48211	EMD-48212	EMD-48213
Number of molecular projection images in map	22,488	26,470	32,495	16,523	14,422	19,846	15,599	16,158
Symmetry	C1	C1	C1	C1	C1	C1	C1	C1
Map resolution (FSC 0.143; Å)	4.2	4.0	4.0	4.5	4.5	4.2	4.4	4.4
Map sharpening B-factor ($Å^2$)	-72	-67.6	-70.3	-85.1	-85.9	-70	-85.5	-88

Table S4. Cryo-EMPEM refinement parameters for ModWu pAbs

**Map**	**ModWu-RBD1**	**ModWu-RBD2**	**ModWu-NTD1**	**ModWu-NTD2**	**ModWu-NTD3**	**ModWu-NTD4**	**ModWu-NTD5**	**ModWu-NTD6**
EMDB	EMD-48188	EMD-48189	EMD-48190	EMD-48192	EMD-48193	EMD-48194	EMD-48195	EMD-48196
Number of molecular projection images in map	18,968	26,146	16,741	18,469	15,750	29,024	22,055	19,865
Symmetry	C1	C1	C1	C1	C1	C1	C1	C1
Map resolution (FSC 0.143; Å)	3.2	3.2	3.4	3.7	3.4	3.7	3.8	3.4
Map sharpening B-factor ($Å^2$)	-46.8	-56.1	-66.2	-6.25	-42.4	-3.1	-84.8	-44.4

Table S5. Cryo-EMPEM refinement parameters for ModDe pAbs

**Map**	**ModDe-RBD1**	**ModDe-NTD1**
EMDB	EMD-48197	EMD-48198
Number of molecular projection images in map	15,351	8,325
Symmetry	C1	C1
Map resolution (FSC 0.143; Å)	3.6	4.3
Map sharpening B-factor ($Å^2$)	-55.4	-56
